# Monitoring Systems and Numerical Models to Study Coastal Sites †

**DOI:** 10.3390/s19071552

**Published:** 2019-03-30

**Authors:** Elvira Armenio, Mouldi Ben Meftah, Diana De Padova, Francesca De Serio, Michele Mossa

**Affiliations:** 1 Department of Civil, Environmental, Land, Building Engineering and Chemistry (DICATECh), Polytechnic University of Bari (Italy), via Orabona 4, 70125 Bari, Italy; mouldi.benmeftah@poliba.it (M.B.M.); diana.depadova@poliba.it (D.D.P.); francesca.deserio@poliba.it (F.D.S.); michele.mossa@poliba.it (M.M.); 2 CoNISMa, National Interuniversity Consortium of Marine Sciences, Piazzale Flaminio 9, 00196 Roma, Italy

**Keywords:** monitoring station, numerical modelling, current circulation, oil spilling

## Abstract

The present work aims at illustrating how the joint use of monitoring data and numerical models can be beneficial in understanding coastal processes. In the first part, we show and discuss an annual dataset provided by a monitoring system installed in a vulnerable coastal basin located in Southern Italy, subjected to human and industrial pressures. The collected data have been processed and analysed to detect the temporal evolution of the most representative parameters of the inspected site and have been compared with recordings from previous years to investigate recursive trends. In the second part, to demonstrate to what extent such type of monitoring actions is necessary and useful, the same data have been used to calibrate and run a 3D hydrodynamic model. After this, a reliable circulation pattern in the basin has been reproduced. Successively, an oil pollution transport model has been added to the hydrodynamic model, with the aim to present the response of the basin to some hypothetical cases of oil spills, caused by a ship failure. It is evident that the profitable prediction of the hydrodynamic processes and the transport and dispersion of contaminants strictly depends on the quality and reliability of the input data as well as on the calibration made.

## 1. Introduction

Coastal areas are highly vulnerable because they are exposed to both natural hazards, including flooding, storm impacts, sea-level rise, and coastal erosion, and to anthropic activities such as urbanization, industrialization and transportation on the other hand. Nearshore regions provide several important functions, with high monetary value, managed by different stakeholders, e.g., fishing, aquaculture, leisure and tourism, water supply, wastewater treatment, construction, harbours and energy production. Furthermore, they are often a valuable environmental heritage, thus requiring proper safeguarding and enhancement, to be achieved by means of sustainable development, education and environmental protection [1,2,3].

In recent years, considerable attention has been paid to the preservation of coastal basins and inlets, which are especially vulnerable to human interventions and climate change because of their semi-enclosed shapes and lagoon characteristics. They represent a noteworthy natural resource, characterized by dynamic ecosystems in which natural and anthropic processes interact. Thus, their massive exploitation due to dense population and industrial activities could alter their geomorphological, physical and biological features, causing heavy pollution phenomena [3]. Consequently, both politicians and coastal managers are strongly required to strive to understand the evolution of the coastal system and guaranteeing its preservation.

To achieve this goal and support the environmental policy, the monitoring activity is one of the most appropriate tools in controlling and preventing the response of coastal areas to human actions and natural hazards [4]. This is more necessary in sensitive coastal sites characterized by a plurality of pressure factors, such as urban and industrial discharges or intense naval traffic or large harbours and military arsenal settings, where accidental releases of crude oil, gas and chemical products could take place. Accidental and illegal oil pollution constitutes a major threat to the marine environment and the risk of coastal oil spills dramatically increases.

The most common nautical accidents occur due to sinking or foundering, grounding, structural failure, scuttling, by contact or collision, explosion or fire, or after disappearance or abandonment. The discharge from oil pipelines, oil platforms, and vessels also causes significant damage to the marine environment and coastal areas. As shown by [5] and [6], marine oil spills may lead to serious environmental disasters, often with significant long-term ecological impacts on the coastal environment and detrimental consequences on the socio-economic activities of the area.

Successful responses to oil spill events can be achieved, provided that detailed information on type and volume of the escaped oil is promptly available to field operators. With information on the oil slick location, extent, thickness, and expected drift direction, the response team can plan effective countermeasures to mitigate the effects of devastating pollution on the marine environment [7]. To facilitate the decision-making process, it is possible to obtain very positive support for oil spill monitoring thorough the joint use of oil drift models, remote-sensing observations and measurement stations. It is worth noting that remote sensing techniques, widely and effectively adopted for oil spill monitoring scopes [7] are based on both microwave observations given by synthetic aperture radar (SAR) and satellite optical imagery. However, these types of data are not always available for the target sites and for the desired time period. In such cases, at least the coupling of advanced monitoring technologies and numerical models is required [7]. Furthermore, for consistent and accurate results, it is essential to run numerical models previously implemented and calibrated with reliable field data.

Since the 1960s, numerous oil spill models have been developed by various organizations, companies, and researchers, to simulate weathering processes and forecast the fate of oil spilled, in terms of providing valuable support to both contingency planners and pollution response teams.

There are two categories according to the Industry Technical Advisory Committee (ITAC) for oil spill response. One category, known as oil weathering models, estimates how oil properties change over time under the influence of current and wind advection, but does not predict potential migration of the slick [8]. The second category includes trajectory or deterministic models, stochastic or probability models, hind cast and three-dimensional models. In addition to predicting weathering profiles, these models estimate the evolution of a slick over time [9,10].

Some of the oil spill models that are currently available are: General NOAA Operational Modeling Environment (GNOME) [11], MEDSLIK-II [12], SeaTrackWeb (STW) [13], Model Oceanique de Transport d’ Hydrocarbures (MOTHY) [14], DieCAST-SSBOM (Shirshov-Stony Brook Oil spill transport Model) [15], COastal Zone OIL spill model (COZOIL) [16], POSEIDON Oil spill model [17].

Specifically, in the present study, we have addressed the DHI MIKE 3 FM Oil Spilling Model [18], which belongs to the second category of the models described above. This numerical model has effectively demonstrated its ability to accurately analyse oil spill events [19,20,21,22,23,24,25,26] provided that an adequate calibration for the hydrodynamic module is performed first. As shown by [27] for the Fu Shan Hai oil spill accident in the Danish waters, the DHI Oil Spilling model, forced by not calibrated currents, provided a poor performance.

In this paper, we have focused our interest on a very vulnerable coastal site in southern Italy, the Mar Grande basin. First, an extensive set of monitored data of winds, waves and currents is presented and discussed, recorded in this semi-enclosed coastal basin in the year 2015. These data have been then used to calibrate the DHI 3D hydrodynamic flow model [18]. After this, some scenarios of oil spreading arising from a continuous 72-h spill of crude oil from a ship failure, for selected winter and summer periods in 2015, have been hypothesized and examined. The MIKE 3 FM Oil Spilling Model [18] based on dispersive processes, has been added to the hydrodynamic module, to simulate the possible oil spreading.

## 2. Materials and Methods

### 2.1. Study Site and Monitoring Station

**Figure 1:**
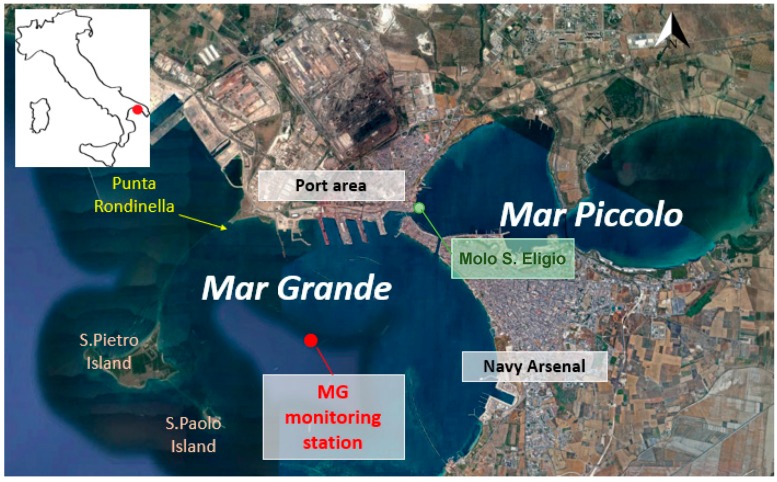
Map of Mar Grande basin and location of the monitoring station MG.

The Mar Grande is a coastal basin with a typical round shape, located in the inner northeastern area of the Ionian Sea, in Southern Italy (Figure 1). Its total surface area is about 35 km^2^, while its maximum depth is about 35 m in its central part. The Mar Grande is connected to a smaller semi-enclosed basin, named Mar Piccolo, formed in turn by two embayments. The Mar Grande and the Mar Piccolo basins are joined by means of an artificial channel, i.e., the Navigable Channel, and a natural one, i.e., the Porta Napoli Channel. As shown in Figure 1, on its western side the Mar Grande is limited by two small isles (S. Pietro and S. Paolo) called Cheradi, connected each other by a long breakwater. The northwestern opening between the mainland and the Cheradi Isles is named Punta Rondinella and is about 100 m long, while the southwestern one between the Cheradi Isles and the mainland is about 1400 m long. All the port activities are located along the northern coast of the Mar Grande, while the Naval Arsenal is located along the southern coast. The urban centre is located along the eastern coast (Figure 1). Over the years, the basin has been subjected to a heavy polluting charge, affected by different outflows from sources of civil, military and industrial origins, which are authorized and monitored only in some cases.

To start collecting some information about the physical and hydrodynamic state of the basin, a meteo-oceanographic station was installed in its central area (here named for brevity MG station), in December 2013, at the geographical coordinates 40°27.6’ N and 17°12.9’ E, where the local depth is around 23.5 m. The devices were settled in the frame of the Italian Flagship Project RITMARE [28] and the entire system is managed by the research unit of the Polytechnic University of Bari–Laboratory of Coastal Engineering (LIC). The station is equipped with many instruments, including a bottom mounted Acoustic Doppler Current Profiler (ADCP), a multidirectional wave array (both by Teledyne RD) and a weather station (by Met Pack). Other scalar parameters, such as temperature, salinity, chlorophyll, are also measured at five meters below the sea surface [29].

### 2.2. Monitoring System

The monitoring system’s configuration of the MG station is based on a telemetry data transmission technique, to acquire field data and transmit them in real time, ensuring high standards of efficiency and precision even over considerable distances. Its network architecture has been implemented to acquire measurements and communicate them to receivers, thus allowing a continuous exchange and update of information. The monitoring station is composed of various sensors and devices connected each other to implement and integrate the data management network. Through a client-server system and relying on a protocol architecture, it allows the sharing of the acquired measurements. In particular, the monitoring system configuration performs the following activities:

(1) continuous detection of the measured environmental parameters using suitable sensors and devices installed in the station;
(2) remote pre-processing of raw data for decoding from binary files in a user-friendly format to be easily managed by users;
(3) wireless data transmission via GSM/GPRS devices to connect the MG station to a Data Acquisition Center;
(4) data storage on a dedicated server on which authorized users can visualize and download data also by remote connection.

**Figure 2:**
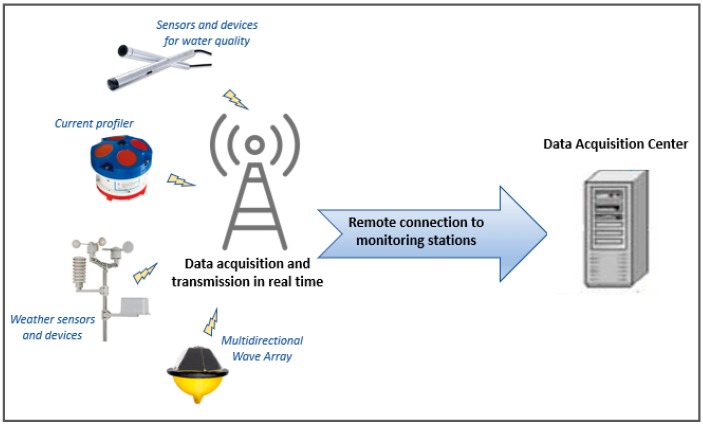
Conceptual scheme of the monitoring system MG.

Figure 2 displays the conceptual configuration of the monitoring station. All data measured by the current profiler, the weather sensors and the multidirectional wave array are both stored onsite, available for remote access queries to check and display their status in real time, and transmitted in real time to the Data Acquisition Center, where it can be downloaded, archived and processed. 

More specifically, the sensors are all connected to an autonomous data acquisition unit, i.e., a datalogger (named LISC), which acquires data from 12 serial ports and 16 analog channels, which allows: (i) data processing onsite and in real time, (ii) remote control and (iii) data download. The datalogger processing, demanding in computing and energy-supply terms, is managed by a software program (MARLIN) installed in a suitable device, which communicates with the LISC at the end of each measurement for a short time window, just necessary to get the raw data and send back the processed ones. The datalogger can be remotely queried by means of a cellular modem 3G, connected to it through a serial port and equipped with a stack TCP/IP to send data on the web cloud. In this way, the communication from the remote system to the devices and vice versa is possible, managed by a proper software (ROCS). The ROCS software can call the LISC datalogger both automatically and manually, permitting to download the acquired data and to check or change the acquisition parameters. Configurations and data from the managed devices are all contained in a relational database, which is the ROCS core, and can be exported in binary raw data or ASCII files, according to user needs. This database is constantly updated. Depending on the type of data, a table is filled by specifying the date and time of recordings. Any anomalies or malfunctions are identified by a specific code.

### 2.3. Analysis of Monitored Data

**Figure 3:**
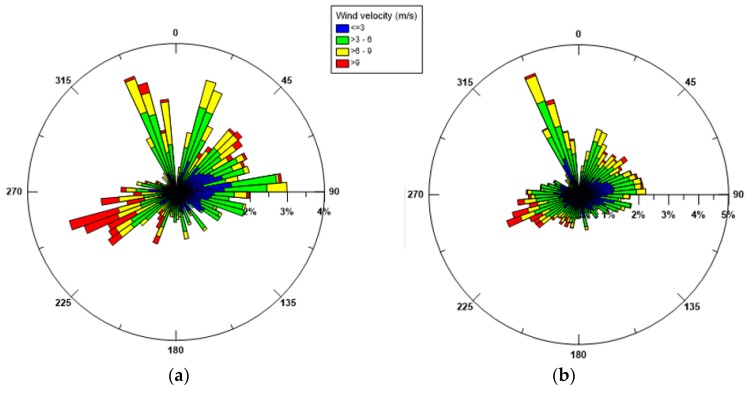
Wind polar diagrams for 2015: (**a**) winter; (**b**) summer. Incoming wind directions are shown.

The wind data for the whole year 2015 have been processed. To identify the predominant seasonal trends, the rose plots referring to the winter and summer period are shown respectively in Figure 3, based on their incoming directions. During the winter period (January–March), the most frequent winds come from NNW, NNE and WSW. Considering the location of the MG station, WSW winds, which are also the most intense ones (intensities >9 m/s), are significant, because they come from the open sea where they originate wind waves on longer fetches. Moderate (3–6 m/s) and high (6–9 m/s) wind intensities are observed along the other directions. During the summer period (July–September), the most frequent winds come mainly from NNW with peak velocities in the high range 6–9 m/s. Compared to these, all the winds coming from different directions are more occasional and almost weak. The wind distribution observed in 2015 replicates the records of the winds measured in both the years 2014 and 2016 [30], especially referring to the winter period, while, for the summer season, the 2015 data do not show significant winds from SE (Scirocco), which on the contrary are more evident in 2014 and 2016 [30].

**Figure 4:**
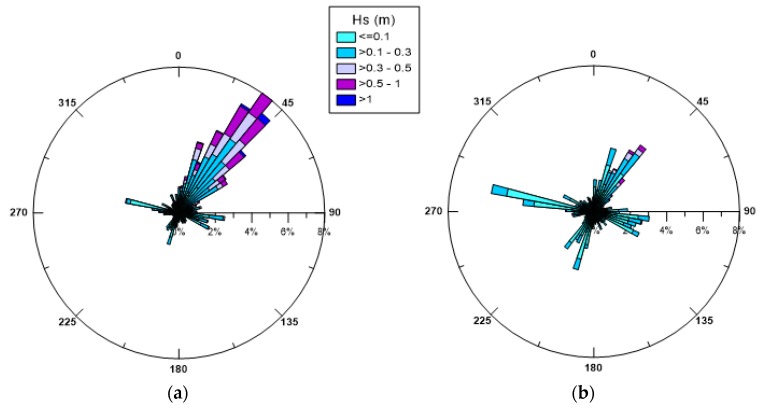
Seasonal trend of the significant wave heights (in m): (**a**) winter 2015; (**b**) summer 2015. Wave propagation directions are shown.

Figure 4 displays the polar plots of the significant wave heights *Hs* monitored during the year 2015, for the winter and summer period respectively, where the directions of wave propagation are shown. The lowest waves are observed in the summer period as expected. In both seasons, a well-defined and evident path is recognized for high waves, which come from SW and propagate towards NE. This wave behaviour confirmed also by the observations of 2014 and 2016 [30], seems consistent with the presence of the wide opening located on the SW boundary of the Mar Grande basin, which allows external swell waves to enter the basin and spread towards the opposite border. The highest values of *H_s_* along this direction are in the range 1.0–1.2 m for both winter and summer cases, although in summer the occurrence of these values is much rarer and generally low waves prevail along all directions (<0.3 m).

**Figure 5:**
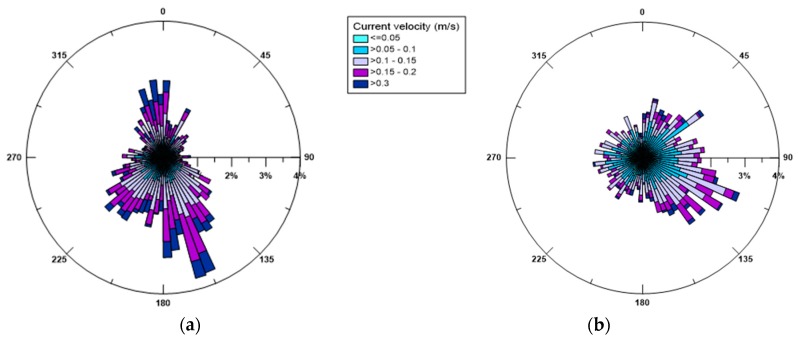
Seasonal currents (in m/s) near the sea surface in 2015 (**a**) winter and (**b**) summer. Direction of current propagation is shown.

In Figure 5 the seasonal distribution of the superficial currents is shown, as measured at a depth of 2 m from the sea surface to disregard the possible influence of waves. Variability in the current direction is evident and especially reflects the variability of the blowing winds. The most frequent and intense surface currents are consistent with the most dominant winds, in fact both in winter and in summer they are mainly directed towards SE and SSE. This observation confirms what already pointed out by De Serio and Mossa [29,30], that is winds blowing from land do not have a direct effect on the origin of sea waves, but rather seem to drive the surface current.

**Figure 6:**
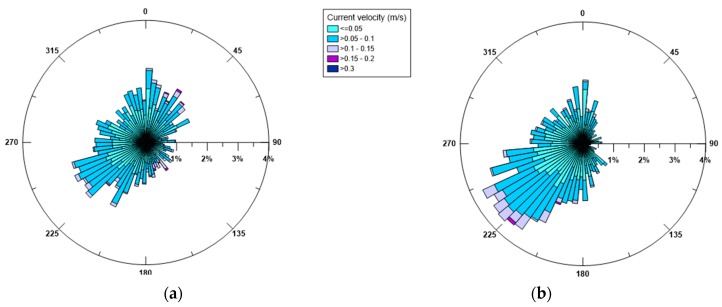
Seasonal currents (in m/s) near the bottom in 2015 (**a**) winter and (**b**) summer. Directions of current propagation is shown.

Analogously, Figure 6 illustrates the polar diagrams of the currents measured near the bottom, for both seasons. The trend observed confirms what was also recorded in 2014 and 2016. In fact, the currents appear to have a preferred direction and tend to converge towards the SW opening of the basin which thus exerts a sort of topographical control [29,30]. By comparing the surface currents with the bottom ones, the highest values (>0.3 m/s) are always noted near the surface, rather than near the bed, where values in the range 0.05–0.1 m/s prevail. Thus, the effect of the wind shear stress, which is the principal driver of the surface circulation, is gradually lost along the water column, and rather at the bottom the currents are controlled by topography.

From the comparison with previous results [29,30], we can note that the behaviours of winds, waves and both superficial and near bottom currents are annually recursive, showing repeating features, remarkably typical for the two examined seasons.

## 3. Numerical Modelling

### 3.1. Calibration

**Figure 7:**
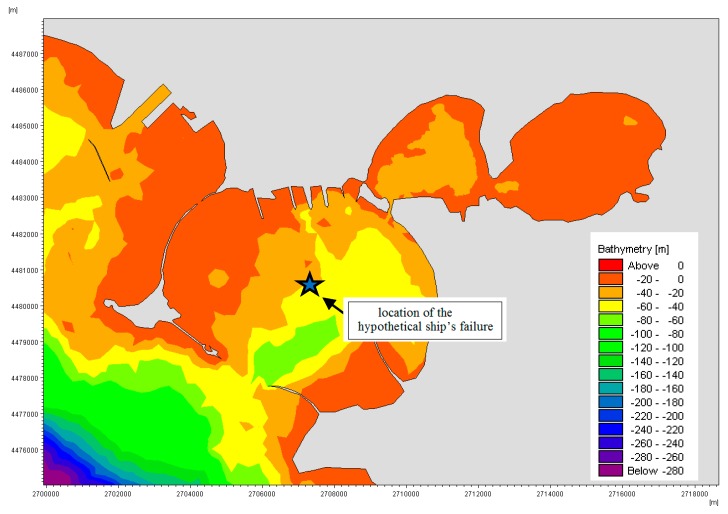
Computation domain used for the numerical simulations. The location of the hypothetical ship’s failure is also shown. UTM coordinates used.

The available field data described above have been used to implement and calibrate the 3D hydrodynamic numerical model MIKE 3 FM HD produced by the Danish Hydraulic Institute (DHI) [15]. A finite mesh of 7235 triangular elements with ten vertical layers has been used (Figure 7). To improve the numerical approach and model more realistic conditions, the hydrodynamic simulations have been carried out in baroclinic mode, with temperature and salinity vertical profiles extracted by the Mediterranean Sea Physics Reanalysis model, characterized by a horizontal grid resolution of 1/16° and by 72 unevenly spaced vertical levels. Moreover, the simulation has been forced at the sea open boundary by the time varying water levels measured at S. Eligio pier (Figure 1) by the National Institute for Environmental Protection and Research (ISPRA). The sea surface wind field has been deduced by the atmospheric model described in [2] and is variable in space and time

The turbulent closure model used within MIKE 3 FM HD model relies on the *k-ε* formulation for the vertical direction [31] and on the Smagorinsky formulation for the horizontal direction [32]. The Smagorinsky coefficient has been assumed uniform in space and temporally constant, equal to 0.6. According to the sensitivity analysis shown by De Padova et al. [28], the simulation has been performed by adopting a seabed roughness equal to 0.1 m.

Based on earlier studies [26,28], the wind drag coefficient *C_d_* has been considered as the calibration parameter to which the model results are most sensitive. Therefore, it has been tuned, switching among different values. We have deduced that the most suitable value of *C_d_* to get the best match between modelled and measured data is 0.02. The magnitude of this drag coefficient is one order higher than in most of the cases studied, but it is worth noting that lower magnitudes are typical of circulation studies involving oceanic sites or large seas, where the action of the wind is very strong. On the contrary, near coastal sites and in confined seas, where action of the wind is weaker, an increased *C_d_* is needed to transmit effective wind stress to surface currents [29].

**Figure 8:**
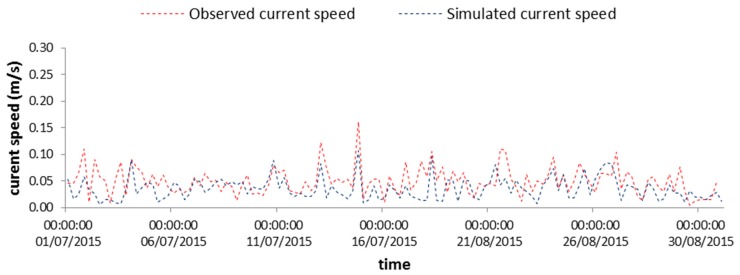
Comparison between time series of observed and simulated current speed at the selected point.

The comparison between modelled and real current velocities has been made for the point where the MG station is located, at a depth of 5 m from the surface, to disregard the possible influence of surface waves on current measurements. For the same reason, the month of July 2015 has been chosen for comparison, because it is characterized by the lowest recorded waves. Figure 8 shows the time series of the computed and observed current intensities. A good similarity between the modelled and measured currents is clearly shown. The degree of agreement has been estimated via the index *I_w_* proposed by Wilmott [33], which has been computed. It takes into account the relative error among field and output values and can be equal to 1, at best. In our case it assumes the value 0.72, thus indicating a good reproduction of the measured data by the model in terms of magnitude. Analogously, *I_w_* has been calculated also to evaluate the model performance in terms of velocity directions, providing, in this case, an average value around 0.6, which is still a satisfactory result, especially considering the complexity of the environment reproduced and the simplifications necessarily applied in the modelling.

### 3.2. Oil Spilling Runs

Once verified the validity of the adopted hydrodynamic model, this has been combined with a dispersive module capable of reproducing the spreading of a contaminant, with the aim of evaluating the response of the model when an oil spilling occurs. Thus, the MIKE 3 FM Oil Spilling model by DHI has been implemented to simulate a hypothetical case of the oil spill caused by a ship failure in the central area of the Mar Grande basin (Figure 7). The oil spill model [18] solves the so-called Fokker–Planck equation for suspended oil substances in two dimensions, through the introduction of a consistent random walk particle method. It is solved by the Lagrangian discrete parcel method, while the weathering processes are solved by the Runga–Kutta fourth-order method. The pollutant is divided into discrete parcels and sets of spatial coordinates are assigned to each parcel. It is assumed that these parcels advect with the surrounding water body and diffuse as a result of random processes. The displacement of each Lagrangian particle is given by the sum of an advective deterministic and a stochastic component, the latter representing the chaotic nature of the flow field, the sub-grid turbulent dispersion. Further details on this module are in [18,24,25].

Two simulation runs, denoted as T1 and T2, have been carried out, with the aim of evaluating how the transport of contaminants in this area can be different during the winter and summer seasons respectively. The spill has been modelled as a continuous leakage of oil over a period of 72 h (3 days) starting on 12 December 2015 at 08:00 for test T1 and on 12 July 2015 at 08:00 for test T2. For each simulation the total mass of oil released is equal to 519 tons (rate of 2 kg/s) and the applied oil type is crude oil.

The sea surface wind field has been deduced by the atmospheric model described in [2] and is variable in space and time. There has been a considerable dispute among modellers about the best choice for the values of the wind drift factor and the wind-drift angle to be used. In fact, it depends on the physical process of the problem and the desired computational efficiency. Most of the models have used a value of around 3% for the former and a value between 0° and 25° for the latter [34,35,36,37]. In this study we have employed the well-established 3% wind factor and a wind drift angle equal to 20° also in accordance with what obtained from our previous study [26]. The oil spill model has been driven by the outputs of the calibrated hydrodynamic model MIKE 3FM, respectively for T1 and T2 cases.

## 4. Results and Discussion

**Figure 9:**
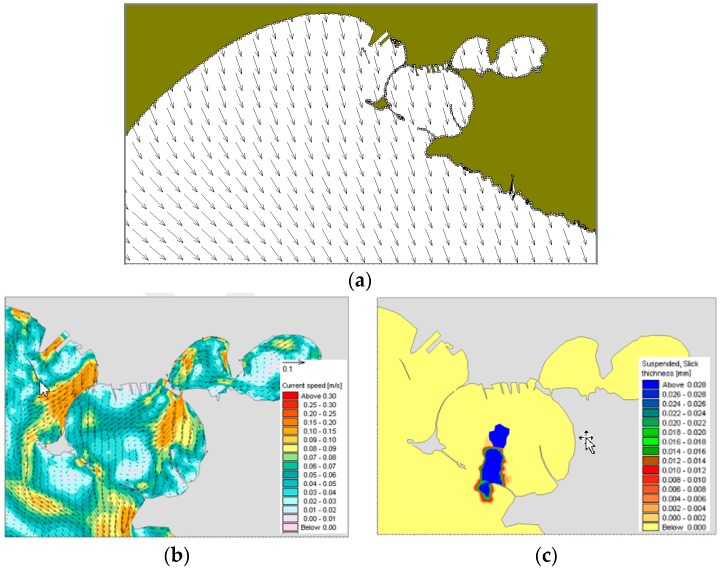
Test T1. Dec. 12 at 18:00. (**a**) Model wind fields; (**b**) Surface current field; (**c**) Oil slicks.

**Figure 10:**
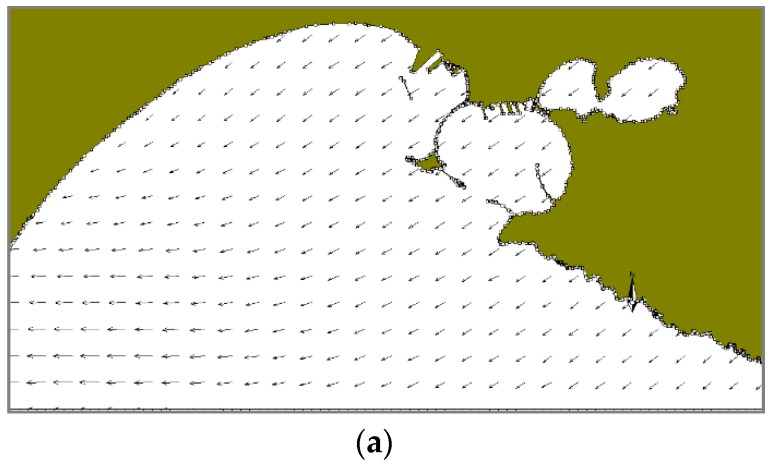

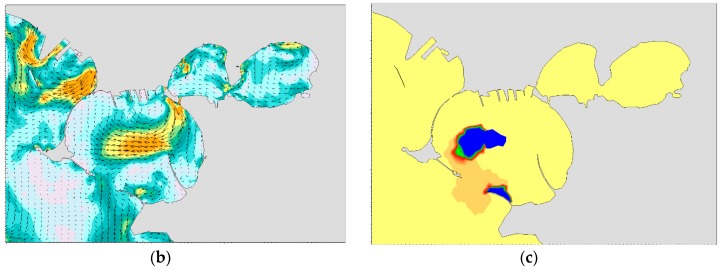
Test T1. Dec. 13, at 18:00. (**a**) Model wind fields; (**b**) Surface currents; (**c**) Oil slicks. Legends as in Figure 9.

**Figure 11:**
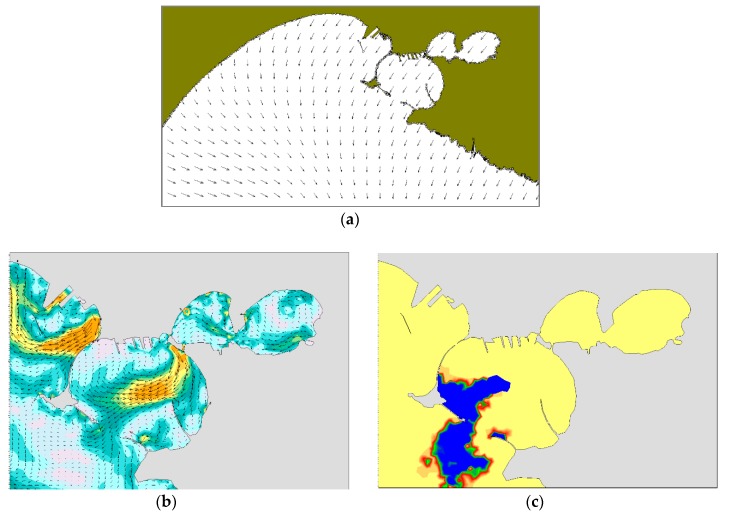
Test T1. Dec. 14, at 18:00. (**a**) Model wind fields; (**b**) Surface currents; (**c**) Oil slicks. Legends as in Figure 9.

In the winter case (T1), the selected period of the run is characterized by mainly NW and NE winds. At 18:00 on December 12, i.e., 10 h after the oil spilling started, NW winds are quite uniformly distributed on the whole domain (Figure 9a). At the same time, there is a superficial circulation mainly outflowing from the Mar Grande basin (Figure 9b) and the oil slick is transported from the centre of the basin towards the SW opening, elongated and directed towards the open sea. At 18:00 on December 13 (Figure 10), the oil slick has changed its orientation and seems more confined to the western boundary, driven by westwards surface currents and the wind from NE. At 18:00 on December 14, due to northerly winds and easterly currents, the oil slick widens along the western boundary and spreads outside the Mar Grande (Figure 11).

**Figure 12:**
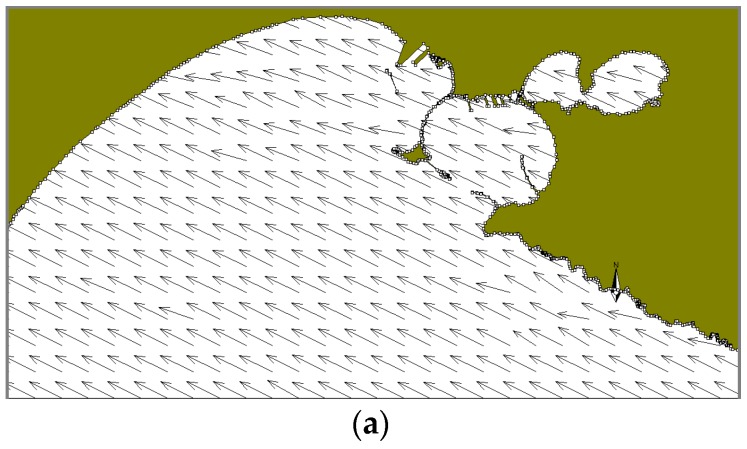

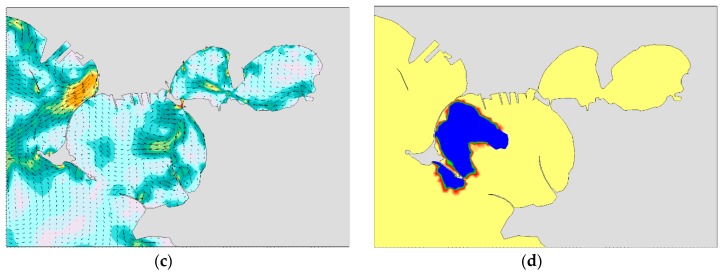
Test T2. July 13, at 00:00. (**a**) Model wind fields (**b**) Surface currents; (**c**) Oil slicks. Legends as in Figure 9.

**Figure 13:**
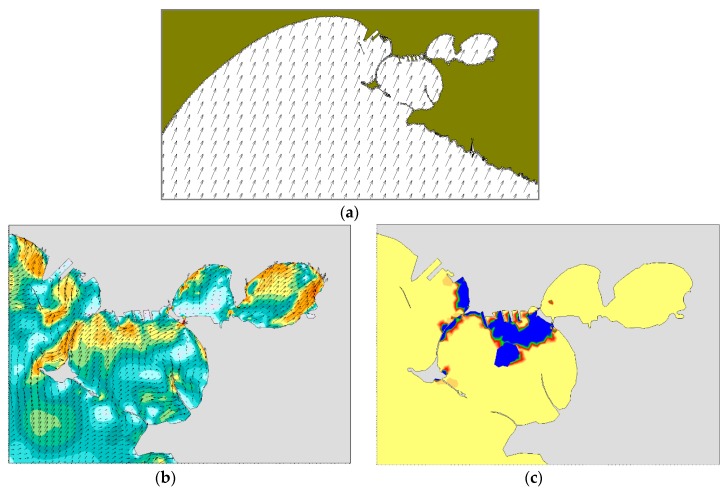
Test T2. July 13, at 12:00. (**a**) Model wind fields (**b**) Surface currents; (**c**) Oil slicks. Legends as in Figure 9.

**Figure 14:**
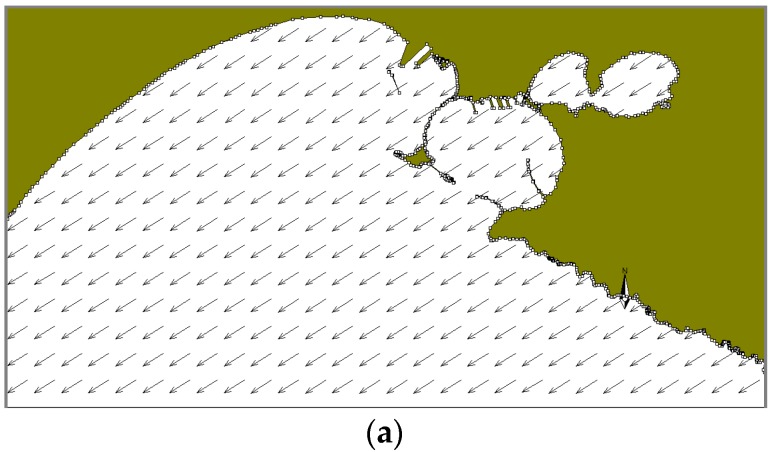

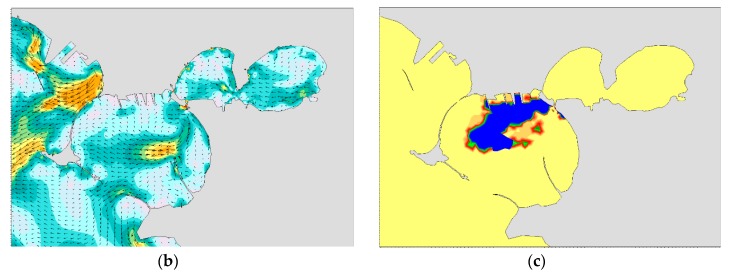
Test T2. July 14, at 00:00. (**a**) Model wind fields (**b**) Surface current field from; (**c**) Oil slicks. Legends as in Figure 9.

In the summer case (T2), the selected period of the run is characterized by very variable winds. After the first day (July 12) with currents directed outside the Mar Grande basin under the influence of NE winds, at 00:00 on July 13 (Figure 12) the wind direction changes and SE winds induce in the central part of the domain a clockwise trend, with increasing flow intensity. Consequently, the oil slick, is transported towards the NW border. At 12:00 on July 13, SW winds blow (Figure 13) and an intense northwards flow is observed along the coast of San Pietro Isle and along the northern coast of the basin. The oil slick is moved and spreads towards the northern border of the basin, in the port area. On July 14 at 00:00, the winds change direction again, becoming NE winds (Figure 14), inducing the formation of weak currents along the northern border, a cyclonic vortex in the central part of the domain and a weak outflow towards the open sea. Thus, in this case, the oil slick tends to return to the centre of the basin.

For both tests T1 and T2, the main results of the oil pollution transport model show that the oil slick is primarily moved by the action of surface currents, whose variability in turn depends strongly on winds, rather than on other forces. The intensity and direction of the surface currents drive the dispersion of the oil slick, determining its shape and size. In particular, in test T1, predominantly NW and NE winds induce in the basin an intense outward current or a clockwise one feeding an outflowing branch. They both allow the oil slick travelling throughout the basin, directed outside. In test T2, under the action of weaker winds with a varying direction (clockwise from South to North), a clockwise trend is observed in the surface circulation. As a result, the northern and western coasts of the Mar Grande are seriously exposed to an oil pollution load. This increases the hazard in such areas, which are already vulnerable, under the polluting pressure due to the presence of the port. Moreover, both in winter (T1) and in summer (T2), the eastern coast of the Cheradi Isles (Figure 1), where some important natural species such as *Poseidonia oceanica* have been observed [38,39,40], is reached and affected by oil pollution. Strong and negative impacts occurring on the marine environment, like this one, could be monitored and even predicted by a trustworthy numerical simulation. It is evident that the possible performed scenarios and the output of the oil pollution transport model can be considered reliable only when reliable input and boundary conditions are used. This further proves the need to adopt field data of high quality in the use of the model. Operating in this way permits a profitable coastal management and intervention in the event of accidents.

## 5. Conclusions

The paper has shown to what extent the joint use of data from monitoring stations and numerical models is necessary and useful in a very vulnerable coastal site. Specifically, we have proved that this synergy is possible if based on high quality available field data. In fact, data sets including many different parameters, which, assessed continuously and for a sufficiently long time, are needed to produce consistent hydrodynamics of the target area.

Firstly, the extensive set of monitored data of winds, waves and currents recorded in the Mar Grande basin, referring to the year 2015, has been examined. We have deduced that during the winter period, the most frequent winds are from NNW, NNE and WSW, with WSW winds being the most intense ones, coming from the open sea where they originate wind waves on longer fetches. During the summer period, the most frequent winds come mainly from NNW, with peak velocities in the high range 6–9 m/s. The polar plots of the significant wave heights at the monitoring station reveals that in both seasons there is an evident path for the highest waves, reaching the station from SW and propagating towards NE, due to the presence of the wide opening located on the SW boundary of the basin. The distribution of the superficial currents is consistent with the most dominant winds, in both winter and summer. Near the bottom, the measured currents have a preferred direction and tend to converge towards the SW opening, which thus exerts a sort of topographical control.

In the second part of the paper, the MIKE 3D FM model has been calibrated with the examined field data, by tuning the wind drag coefficient. These results highlight that the calibrated model can detect and reproduce the main features of the circulation structure in the target area. Successively, once recognized that the hydrodynamic pattern of the basin is the first necessary step for further investigations, the MIKE 3D FM Oil Spilling model has been implemented to simulate a hypothetical case of an oil spill caused by a ship failure in the central area of the Mar Grande. The oil spill model has been driven by the outputs of the calibrated hydrodynamic model MIKE 3FM. Two simulation runs have been carried out, with the intent to assess the different way in which the contaminant is transported in this area during the winter and summer seasons of 2015. For both tests, the principal results show that the oil slick is governed primarily by the action of surface currents, in turn driven by the winds. In the winter case, with predominantly NW and NE winds, it travels the basin directed outside. In the summer case, under the action of winds with varying direction, the northern and western coasts of the Mar Grande are seriously exposed to an oil pollution load. As well, the eastern coast of the Cheradi Isles, characterized by the presence of important natural species, such as Posidonia Oceanica, is dramatically reached by oil pollution.

The proposed scenarios highlight the necessity to properly reproduce hydrodynamics in the basin, to provide useful information and predictions. This can be achieved through the joint use of high-quality field data and numerical models.
